# Supplementary material for: Lenvatinib-Loaded Poly(lactic-co-glycolic acid) Nanoparticles with Epidermal Growth Factor Receptor Antibody Conjugation as a Preclinical Approach to Therapeutically Improve Thyroid Cancer with Aggressive Behavior
Source: Biomolecules. 2023 Nov 13;13(11):1647. doi: 10.3390/biom13111647 (PMC10668968; doi:10.3390/biom13111647)
Supplement: Supplementary file 1 [file biomolecules-13-01647-s001.zip › biomolecules-2663375-supplementary.pdf]

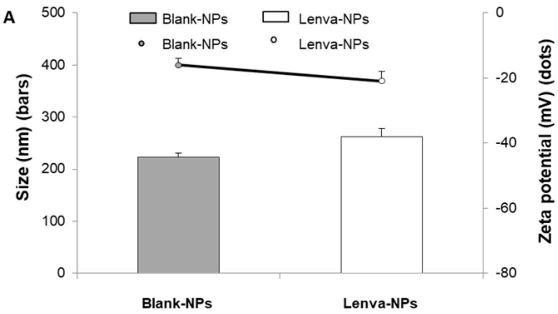

B

Nanoparticles	Dispersity	SD
Blank-NPs	0.12	0.05
Lenva-NPs	0.19	0.01

Figure S1. Physicochemical characterization of blank-NPs and lenva-NPs. (A) Size (bars), and zeta potential (dots), (B) Dispersity index and standard deviation values of blank-NPs and lenva-NPs. Each value represents the mean ± standard deviation of three measurements.

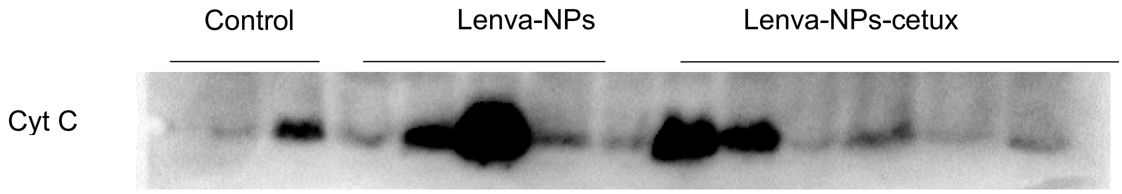

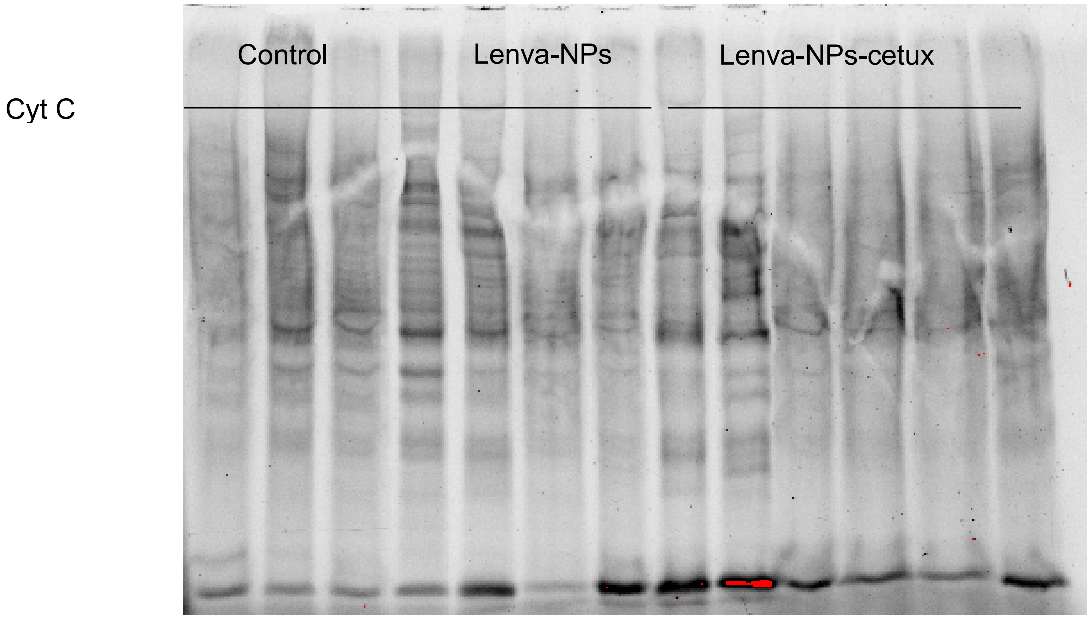

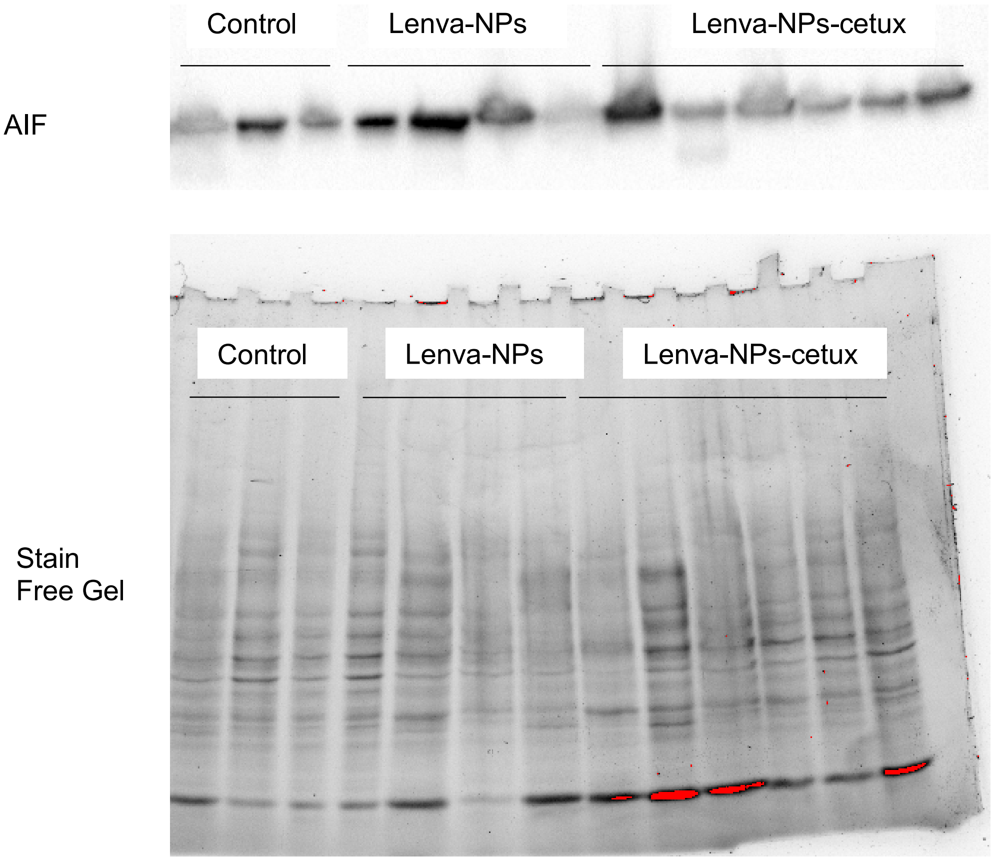

Figure S2. Original Western Blots of apoptosis markers (Cyt C and AIF) of Figure 10.
